# Shrinking Lung Syndrome in a Young Female: A Rare Pulmonary Manifestation of Systemic Lupus Erythematosus

**DOI:** 10.7759/cureus.24320

**Published:** 2022-04-20

**Authors:** Mohamed Ramzi Almajed, Mark S Obri, Shazil Mahmood, Zachary D Demertzis

**Affiliations:** 1 Internal Medicine, Henry Ford Hospital, Detroit, USA; 2 Internal Medicine, Henry Ford Health System, Detroit, USA

**Keywords:** pulmonary function tests, interstitial lung disease, restrictive lung disease, systemic lupus erythematosus, shrinking lung syndrome

## Abstract

Shrinking lung syndrome (SLS) is a rare pulmonary complication of autoimmune conditions. It has been sparsely described in the literature and its pathophysiology remains unclear. SLS is typically reported in patients with a history of systemic lupus erythematosus (SLE) who present with shortness of breath and chest pain associated with breathing. Chest imaging demonstrates no alveolar, interstitial, or pleural abnormalities. Pulmonary function tests (PFTs) are characterized by a restrictive pattern with reduced lung volumes. SLS is a diagnosis of exclusion and there are no validated criteria for the diagnosis. Evaluation requires extensive testing to rule out alternative causes of dyspnea and pleuritic chest pain. In this report, we present a case of SLS in a young African American woman.

## Introduction

Shrinking lung syndrome (SLS) is a rare pulmonary complication of systemic lupus erythematosus (SLE) that has an unknown pathophysiology. Patients typically have a history of autoimmune disease and present with unexplained dyspnea and pleuritic chest pain [1]. Pulmonary imaging is characterized by the absence of radiographic alveolar, interstitial, or pleural abnormalities. Pulmonary function tests (PFTs) support the diagnosis by demonstrating a restrictive pattern with reduced lung volumes [2].

There are no validated criteria for the diagnosis of SLS. It is a diagnosis of exclusion that necessitates extensive workup to rule out other causes of dyspnea and pleuritic chest pain in the appropriate clinical context. The case we report here represents an uncommon diagnosis for a common presentation.

## Case presentation

A 26-year-old African American woman presented to the hospital with a three-week history of shortness of breath. Her medical history was significant for SLE for which she had never received medical therapy. She had developed end-stage renal disease secondary to SLE-associated membranoproliferative glomerulonephritis, had undergone a failed kidney transplant, and had been on intermittent hemodialysis for two years. The patient also had experienced non-ischemic heart failure with a reduced ejection fraction and mild-intermittent asthma, both of which had been medically managed appropriately. Recently, she had started taking increasing doses of opioids for chronic pain.

On presentation to the hospital, the patient reported a two-day history of pleuritic chest pain and a three-week history of shortness of breath that worsened on inspiration, lying flat, and ambulation. Her symptoms improved with leaning forward and while resting. She reported chronic orthopnea and paroxysmal nocturnal dyspnea that were unchanged.

In the emergency department, the patient was found to be tachypneic with lungs clear to auscultation. Arterial blood gas on room air revealed acute respiratory acidosis and oxygen saturation of 99%. Initial investigations showed an elevated D-dimer and erythrocyte sedimentation rate. Troponin, brain natriuretic peptide, and electrocardiogram were unremarkable. Chest X-ray and CT pulmonary angiography revealed no pulmonary embolism and no parenchymal lung disease. She was intubated for worsening hypercapnic respiratory failure and admitted to the medical ICU.

In the ICU, the patient was managed for an asthma exacerbation with intravenous steroids and bronchodilators. Serial chest X-rays remained unremarkable, and a transthoracic echocardiogram revealed a recovered ejection fraction of 63%, normal systolic and diastolic function, and normal right ventricular size and function. After 48 hours of intubation and supportive management, her vital signs and laboratory tests showed improvement and she was extubated successfully.

Despite optimal therapy for asthma exacerbation, the patient continued to endorse dyspnea with fatigability. Blood gases revealed persistent respiratory retention of carbon dioxide with an arterial blood gas showing a chronic respiratory acidosis and an oxygen saturation of 96%. This prompted further investigation into the underlying etiology of her symptoms.

Inpatient pulmonary and rheumatology services were involved, and their initial differential diagnoses included interstitial lung disease, neuromuscular disease, and myopathy. Autoimmune workup was significant for a positive antinuclear antibody (ANA) titer of 1:320 (speckled pattern), low C3, and normal C4 levels. Further testing including aldolase, CPK, SSA/SSB antibodies, and myositis antibodies panel was negative. Electromyography indicated possible chronic myopathy and was negative for a phrenic mononeuropathy; however, MRI and muscle biopsy were negative for a myopathic process.

PFTs performed during the admission revealed a restrictive pattern with reduced lung volumes, reduced diffusion capacity, and reduced respiratory muscle force (Table 1).

**Table 1:** Complete PFTs demonstrate a restrictive pattern with reduced lung volumes that correspond to SLS PFTs: Pulmonary function tests; SLS: shrinking lung syndrome; FVC: forced vital capacity; FEV1: forced expiratory volume in 1 second; SVC: slow vital capacity; FEV3: forced vital capacity in 3 seconds; PEF: peak expiratory flow; FET100%: forced expiratory time; MVV: maximum voluntary ventilation; TLC: total lung capacity; VC: vital capacity; IC: inspiratory capacity; FRC: functional residual capacity; ERV: expiratory reserve volume; RV: residual volume; DLCO: diffusion capacity for carbon monoxide; VA: alveolar volume; IVC: inspiratory vital capacity; Raw: airway resistance; Gaw; airway conductance; PI max: maximal inspiratory pressure; PE max: maximal expiratory pressure

Pulmonary function test	Measure	Units	Patient value	Reference value	Patient’s percentage of the reference value
Spirometry	FVC	Liters	1.77	3.14	56
	FEV1	Liters	1.40	2.72	51
	FEV1/FVC	%	79	86	
	FEV1/SVC	%	78	86	
	FEV3	Liters	1.72	3.05	56
	FEV3/FVC	%	97	97	
	PEF	L/sec	3.00	6.74	45
	FET100%	Sec	6.02		
	MVV	L/min	61	114	54
Lung volumes	TLC	Liters	3.29	4.31	76
	VC	Liters	1.80	3.14	57
	IC	Liters	0.65	1.84	35
	FRC	Liters	2.64	2.47	107
	ERV	Liters	0.73	1.31	56
	RV	Liters	1.49	1.17	128
	RV/TLC	%	45	26	
Diffusing capacity	DLCO	mL/mmHg/min	13.8	23.2	59
	VA	Liters	3.07	4.19	73
	IVC	Liters	1.60	3.14	51
Resistance	Raw	cmH_2_O/L/sec	2.57	1.15	223
	Gaw	L/sec/cmH_2_O	0.389	2.700	14
Respiratory muscle force	PI max	cmH_2_O	73	91	80
	PE max	cmH_2_O	69	156	44

**Figure 1:**
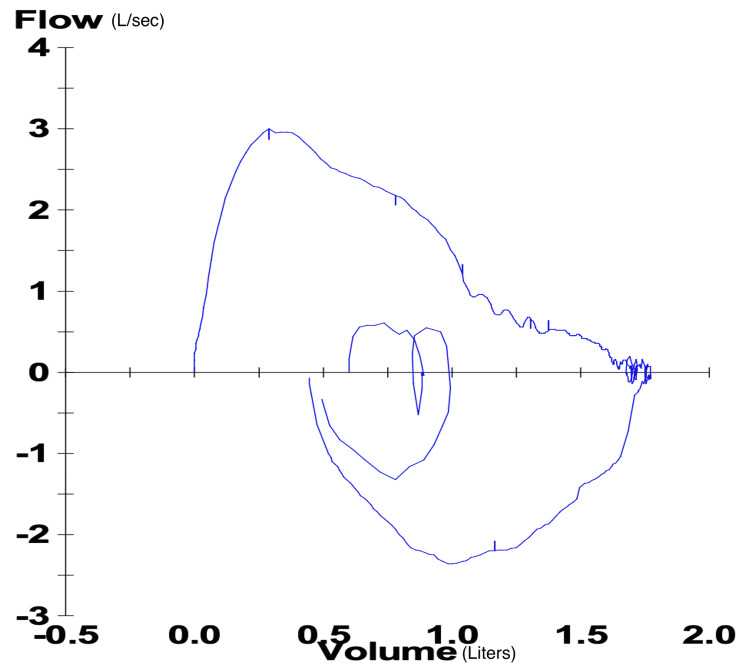
Flow-volume loop shows a restrictive defect with reduced lung volumes, particularly TLC and VC TLC: total lung capacity; VC: vital capacity

**Figure 2:**
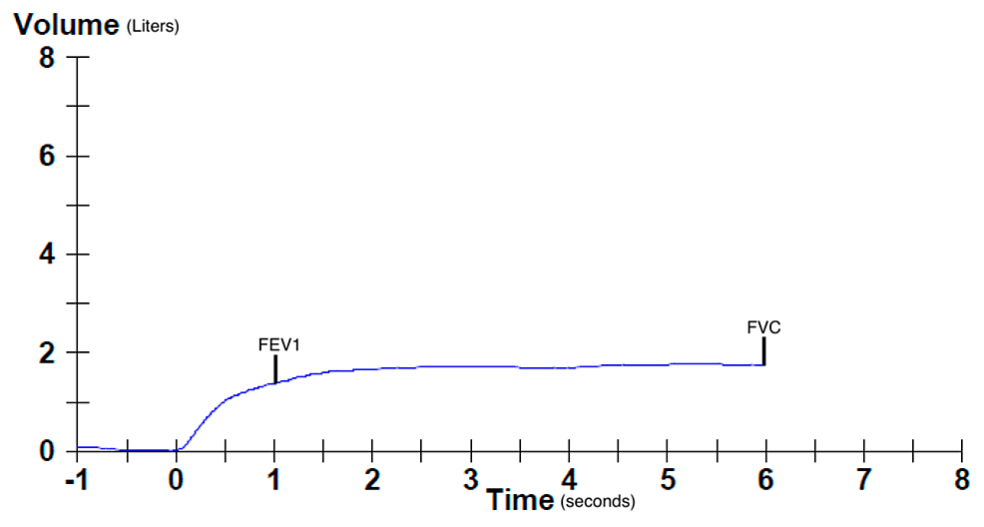
Volume-time curve showed a proportional and significant decrease in the FEV1 and FVC FVC: forced vital capacity; FEV1: forced expiratory volume in 1 second

Flow-volume loop demonstrated a characteristic restrictive deficit (Figure 1). The volume-time curve showed a proportional and significant decrease in the forced expiratory volume in one second and forced vital capacity (Figure 2).

A review of the patient’s chest X-rays revealed a slight elevation of the left hemidiaphragm; this had not been previously seen on her chest imaging and there was no concern for iatrogenic ipsilateral phrenic nerve injury given her lack of exposure to implicated procedures. These clinical findings, in the context of the patient’s SLE, suggested SLS. The patient completed a high-dose course of corticosteroids and was discharged on oral steroids. PFTs were repeated as an outpatient and revealed an increase in lung volumes, indicating a response to treatment.

## Discussion

SLS remains a poorly understood pulmonary manifestation that typically occurs in the setting of autoimmune disease. It has a prevalence of 0.5% to 1.0% and is often unrecognized and underdiagnosed given its rarity and the high clinical suspicion required to identify it [3]. Proposed hypotheses include pleural inflammation and adhesion, phrenic nerve demyelination, diaphragmatic dysfunction, and alveolar surfactant dysregulation [4-6]. It is likely that this syndrome is a manifestation of multiple pathological mechanisms that occur in the setting of a dysfunctional immune response as seen in our patient.

Clinical manifestations of SLS include dyspnea, pleuritic chest pain, and orthopnea; patients typically have no fever, cough, or sputum production [2]. Chest X-ray is unremarkable but may demonstrate unilateral or bilateral elevation of the diaphragm [7]. High-resolution CT is necessary to rule out alternative causes of dyspnea as patients with SLS show no radiographic alveolar, interstitial, or pleural abnormalities [8]. Associated features that have been identified in patients with SLS include positive anti-dsDNA antibodies (in 81% of patients) and positive anti-SSA/Ro antibodies (in 35% of patients). Concomitant involvement of the skin, joints, and kidneys by SLE was commonly seen in these patients [2,7].

PFTs strongly support the diagnosis as all patients have a characteristic restrictive deficit; this is identified by a decreased forced expiratory volume and decreased forced vital capacity. The ratio of these two parameters, denoted by FEV1/FVC, is normal or increased [7]. Moreover, some of these patients have a reduced diffusion capacity (DLCO) and demonstrate findings suggestive of global inspiratory muscle weakness [9-10]. Although a reduced DLCO is commonly interpreted as a sign of interstitial pathology impairing diffusion across from the alveoli to the blood, it can occur in the absence of interstitial pathology. In SLS, a reduced DLCO is attributed to the decreased effective alveolar surface area caused by the lower lung volumes; it can also be attributed to consequential pulmonary hypertension and alveolar surfactant dysregulation [11-12].

An extensive workup is necessary to rule out alternative diagnoses that could explain the symptoms seen in SLS. Neuromuscular pathologies are evaluated by nerve testing and electromyography; phrenic nerve conduction testing is particularly useful. Myopathic pathologies are evaluated by testing for aldolase, CPK, SSA/SSB antibodies, and myositis antibodies panel; MRI and muscle biopsy are more specific and often utilized. These tests were carried out in our case, which allowed for neuromuscular and myopathic processes to be ruled out. Diaphragmatic dysfunction and weakness can present with similar symptoms to SLS in addition to a restrictive deficit on PFTs. It is difficult to assess but can be identified by performing supine PFTs and ultrasonography of the diaphragm to assess its thickness and excursion; this was not performed in our case [13-14].

Management of SLS is dependent on reported outcomes of cases in the literature as there have been no randomized controlled trials carried out to date. Given the predicted autoimmune nature of the syndrome, medications that target the immune response have demonstrated clinical benefits in these patients [1-2]. Corticosteroids in moderate-high doses are the first-line agents and are typically successful in inducing disease remission [7-8]. In refractory cases, adjunctive therapy with immunosuppressive agents such as azathioprine and cyclophosphamide has been effective [15-16]. Other agents, such as rituximab, methotrexate, and mycophenolate mofetil, have been utilized with varying success [17-18]. Adjunctive treatments that promote symptomatic relief include chest physiotherapy, inhaled beta-2 agonists, and theophylline [19-20].

The prognosis of patients who are treated appropriately is good with the majority demonstrating clinical response within weeks to months after the initiation of treatment [2,17]. Serial PFTs are useful in monitoring the response to treatment as treatment responders develop a progressive increase in lung volumes and partial recovery of abnormalities; however, a chronic restrictive defect persists [7]. Although patients with SLS experience significant morbidity and decline in quality of life, mortality seldom occurs as a direct consequence of this syndrome.

## Conclusions

Internists should be aware of SLS and suspect it in at-risk patients with unexplained respiratory symptoms and PFTs showing a restrictive pattern and reduced lung volumes. High clinical suspicion for this condition in the appropriate context is necessary to reach a proper diagnosis, especially in patients with an otherwise inconclusive workup. This case highlights the importance of maintaining a broad differential diagnosis, especially in patients with a history of autoimmune disease.
